# Supplementary material for: A plant-based mutant huntingtin model-driven discovery of impaired expression of GTPCH and DHFR
Source: Cell Mol Life Sci. 2022 Oct 17;79(11):553. doi: 10.1007/s00018-022-04587-6 (PMC9576654; doi:10.1007/s00018-022-04587-6)
Supplement: Supplementary file 2 — Supplementary file2 (PDF 4023 kb) [file 18_2022_4587_MOESM2_ESM.pdf]

Supplementary Information for

A plant-based mutant huntingtin model-driven discovery of impaired expression of GTPCH and DHFR

Chiu-Yueh Hung[1,¶], Chuanshu Zhu[1,2,¶], Farooqahmed S. Kittur[1,¶], Maotao He[1,3,¶], Erland Arning[4,¶], Jianhui Zhang[1], Asia J. Johnson[1], Gurpreet S. Jawa[1,5], Michelle D. Thomas[1,6], Tomas T. Ding[1,*] & Jiahua Xie[1,*]

*Corresponding authors: jxie@nccu.edu (JX); tding@nccu.edu (TD)

This PDF file includes:

Supplementary Text

1. PolyQ63 remodeled root proteome extensively
2. PolyQ63 does not affect root hair initiation
3. DAPs possibly responsible for root growth

References only in Supplementary Text (77-89)

Supplementary Tables

Table S1. Metadata of eight samples and three QC arrangements

Table S7. Peroxidases involved in ROS generation

Table S8. Mitochondrial membrane DAPs involved in transport

Table S9. Mitochondrial DAPs involved in the electron transfer chain, amino acid

metabolism and TCA cycle

Table S10. DAPs involved in the tubular ER network and root hair elongation

Table S11. DAPs involved in ribosome biogenesis and ribosome assembly

Table S12. DAPs involved in regulation of glutathione redox status

Table S13. RNA polymerases involved in transcription

Table S14. Serine/threonine-protein kinases and phosphatases involved in cell proliferation and expansion

Table S15. DAPs involved in folate and one carbon metabolism

Supplementary Figures

Fig. S1. Transformation efficiency and regenerated transgenic plants

Fig. S2. Transgenic plants, leaves, seed pods and seeds

Fig. S3. PCR and RT-PCR results of seven transgenic lines per genetic cassette

Fig. S4. Principal component analysis of quantified proteins

Fig. S5. Selected top 10 GO results of abundance-decreased DAPs

Fig. S6. Selected top 10 GO results of abundance-increased DAPs

Fig. S7. ATP levels of young leaf tissues

Fig. S8. Root hair initiation in induced roots

Fig. S9. Normal root tip structure and DAPs associated with root cell division, elongation or expansion

Fig. S10. Characterization of 4-week-old wide-type non-carrier (NCAR) and R6/2 HD mice

Fig. S11. Original blots of 11 proteins before cropping

Fig. S12. Original blots of GTPCH, DHFR and γ-tubulin in cerebral cortex and striatum tissues of R6/2 and NCAR mice

Other Supplementary Tables in Excel Spreadsheets:

Table S2. Identified 44,888 peptides

Table S3. Identified 7,189 proteins

Table S4. 5,073 proteins quantified by ≥ 2 peptides

Table S5. 1,693 DAPs between $Htt_{ex1}Q63$ and $Htt_{ex1}Q21$

Table S6. GO enriched pathways

Supplementary Text

1. PolyQ63 remodeled root proteome extensively

To get insight into polyQ63-mediated changes in root proteome, quantitative proteomic analysis was performed with $Htt_{ex1}Q63$ and $Htt_{ex1}Q21$ young roots. A total of 44,888 peptides (Table S2) and 7,189 proteins (Table S3) were quantified across all samples (database: ftp://MSV000084557@massive.ucsd.edu) after removing low quality peptides with poor chromatographic peak shape, and scaling of the data to the robust mean across all samples. To determine the quality of quantitative proteomic analysis, both analytical variability and biological variability were analyzed. To assess technical reproducibility, the percentage (%) of coefficient of variation (CV) was calculated for each protein across the three injections of a QC pool that were interspersed throughout the study while %CVs were also measured for each protein across the individual analyses to assess biological variability. The mean %CV of the QC pools was 13.9% for all proteins and 11.5% for proteins quantified by 2 or more peptides (Table

S3). Variability of the biological samples (analytical variability plus biological variability) was 25.6% for proteins or 22.9% for proteins quantified by 2 or more peptides (Table S3). The results of %CV indicate that the technical reproducibility was good, which was much higher than the observed biological reproducibility. These data were reflected in the clustering of the QC samples as visualized by PCA analysis (Fig. S4) while the individual samples clustered based on treatment group. Therefore, these detected proteins were used for their differential expression analysis.

Of these 7,189 proteins, 5,073 of them were further quantified by 2 or more peptides (Table S4). A close examination revealed that abundance of 2,696 proteins was decreased while that of 2,377 proteins was increased (Table S4). When a criterion for higher confidence of identification and quantification with a CV<30% across QC pool replicates, a FDR-corrected $p<0.05$, and the cutoff of ≥1.5-fold changes in abundance was used, a total of 1,693 DAPs were identified between $Htt_{ex1}Q63$ and $Htt_{ex1}Q21$ roots (Table S5). Among them, there were 854 abundance-increased and 839 abundance-decreased DAPs in $Htt_{ex1}Q63$ compared to $Htt_{ex1}Q21$. GO enrichment analysis of these DAPs showed that polyQ63 primarily reduced abundances of proteins involved in translation (GO:0006412, p = 2.94e-06; GO:0000462, p = 1.26e-11), folding (GO:0006457, p = 0.000396), and transport (GO:0015031, p = 4.86e-09), especially from vesicle-mediated ER to Golgi (GO:0006888, p=2.89e-06) (Fig. S5; Table S6). The impairment of protein transport and localization is further supported by affected proteins involved in vesicle (GO:0031988, p=0.000321), ER (GO:0005789, p=7.6e-12), Golgi (GO:0000139, p = 1.91e-06), inner mitochondrial membrane (GO:0098800, p=0.0386), and macromolecular complexes (GO:0032991, p=4.03e-14). When DAPs were categorized based on molecular functions, polyQ63 mainly interrupted the abundances of proteins having activities in the transfer of C_1

units (GO:0016741, *p*=4.22e-07), GTPase binding (GO:0051020, *p*=0.000325), RNA binding (GO:0003723, *p*=1.6e-13), structural constituent of ribosome (GO:0003735; *p*=1e-07) and unfolded protein binding (GO:0051082 *p*=5.74e-06).

Among abundance-increased DAPs, the majority were oxidative stress-related proteins involved in producing reactive oxygen species (ROS) and responding to high ROS levels (GO:0006979, *p*=1.65e-11; GO:0072593, *p*=4.53e-11), as well as enzymes involved in various metabolic processes, such as the carbohydrate metabolic process (GO:0005975, *p*=4.3e-09), hydrogen peroxide catabolic process (GO:0042744, *p*=9.99e-12), and carboxylic acid metabolic process (GO:0019752, *p*=3.71e-06) (Fig. S6; Table S6). Proteins localized at extracellular region (GO:0005576, *p*=1.28e-11) were also significantly enriched. In addition, mitochondrial membrane proteins were enriched (GO:0031966, *p*=0.00464). In term of molecular function, some abundance-increased proteins were enriched in regulating the activities of hydrolases (GO:0016787, *p*=3.42e-07), glutathione transferases (GO:0004364, *p*=4.44e-09), transaminases (GO:0008483, *p*=4.15e-06), oxidoreductases (GO:0016491, *p*=1.06e-26), peroxidases (GO:0004601, *p*=9.66e-14), lyases (GO:0016829, *p*=4.67e-06) as well as proteins with binding activities of ions (GO:0043167, *p*=1e-05), cofactors (GO:0048037, *p*=1.5e-11) and heme (GO:0020037, *p*=3.73e-08). The analysis of these identified DAPs demonstrated that polyQ63 caused widespread remodeling of root proteome.

2. PolyQ63 does not affect root hair initiation

In *Arabidopsis*, root hair initiation begins with the establishment of polarity within epidermal cells [77,78] and whether an epidermal cell can be differentiated into root hair (H) or non-hair (N) cell depends on the absence or presence of an inhibitory pathway consisting of the following

transcription factors GLABRA2 (GL2), CAPRICE (CPC), TRANSPARENT TESTA GLABRA (TTG), GLABRA3 (GL3)/ENHANCER OF GLABRA3 (EGL3) and WEREWOLF (WER) (Fig. S8A) [30]. An epidermal cell becomes H cell when CPC-TTG-GL3-EGL3 complex is formed to inactivate an inhibitory transcription factor GL2 of the final step of H cell formation whereas a cell becomes N cell when WER-TTG-GL3-EGL3 complex is formed to promote GL2 activity [30]. To understand whether the phenomenon of no or less root hairs in induced adventitious roots from Htt_{ex1}Q63 shoots is due to affected root hair initiation or outgrowth, we examined these transcription factors related to root hair initiation in our proteomic data (Table S4). Among them, GL2 was found to be reduced 1.5-fold in Htt_{ex1}Q63 roots compared to Htt_{ex1}Q21 (Fig. S8B; Table S4). Although none of other transcription factors were detected among the DAPs, the decreased abundance of GL2 would benefit or at least not inhibit H cell initiation in Htt_{ex1}Q63 roots, suggesting that polyQ63 might not affect root hair initiation at this stage. Indeed, the adventitious roots were induced having hairs when Htt_{ex1}Q63 shoots were inoculated on the surface of solid maintenance medium (Fig. S8C).

3. DAPs possibly responsible for root growth

A root tip consists of meristematic, transition, elongation and maturation zones based on their characteristic cellular activities (Fig. S9A) [31,79]. Our proteomic data suggest that the following DAPs are possibly responsible for the observed restricted root growth and unorganized root cap structure in Htt_{ex1}Q63 (Fig. S9B and S9C). Firstly, nine DAPs associated with cell division, expansion and elongation [34,80-85] were abundance-decreased (Fig. S9B). Secondly, eight ribosome biogenesis proteins together with a group of ribosome assembly factors, including ATPases, GTPases, helicases, and kinases were also among the abundance-decreased

DAPs (Fig. S9C-I; Table S11). Ribosome biogenesis is tightly regulated and inextricably linked to cell division and growth [86]. Impairment of ribosome biogenesis by mHtt has also been observed in an animal HD model [87] and a N2a cell HD model [23]. Thirdly, as many as 27 glutathione *S*-transferases and 10 glutathione metabolism-related proteins were abundance-decreased DAPs (Fig. S9C-II; Table S12), suggesting that glutathione redox status was altered. In plants, it is also known that altered glutathione redox status does not favor either root growth or root apical meristem maintenance [88]. Fourthly, all seven DNA-directed RNA polymerases were found to be abundance-decreased (Fig. S9C-III; Table S13), as also observed in an animal HD model [87]. Lastly, 11 serine/threonine-protein kinases and phosphatases were among abundance-decreased DAPs (Fig. S9C-IV; Table S14), which are known to be involved in cell proliferation and expansion [89]. Since many of these abundance-decreased DAPs play important roles in root growth and development, they may be responsible for defective root growth and root cap integrity.

References only in Supplementary Materials (77-89):

77. Carol, R. J. & Dolan, L. (2002) Building a hair: tip growth in *Arabidopsis thaliana* root hairs. Philos. Trans. R. Soc. Lond. B Biol. Sci. 357:815-821.
78. Carol, R. J. & Dolan, L. (2006) The role of reactive oxygen species in cell growth: lessons from root hairs. J. Exp. Bot. 57:1829-1834.
79. Anderson, C. T., Carroll, A., Akhmetova, L. & Somerville, C. (2010) Real-time imaging of cellulose reorientation during cell wall expansion in Arabidopsis roots. Plant Physiol. 152:787-796.
80. Burk, D. H., Liu, B., Zhong, R., Morrison, W. H. & Ye, Z. H. (2001) A katanin-like protein regulates normal cell wall biosynthesis and cell elongation. Plant Cell 13:807-827.

81. Wagner, T. A. & Kohorn, B. D. (2001) Wall-associated kinases are expressed throughout plant development and are required for cell expansion. Plant Cell 13:303-318.
82. Burger, C., Rondet, S., Benveniste, P. & Schaller, H. (2003) Virus-induced silencing of sterol biosynthetic genes: identification of a *Nicotiana tabacum* L. obtusifoliol-14α-demethylase (CYP51) by genetic manipulation of the sterol biosynthetic pathway in *Nicotiana benthamiana* L. J. Exp. Bot. 54:1675-1683.
83. Soyano, T., Nishihama, R., Morikiyo, K., Ishikawa, M. & Machida, Y. (2003) NQK1/NtMEK1 is a MAPKK that acts in the NPK1 MAPKKK-mediated MAPK cascade and is required for plant cytokinesis. Genes Dev. 17:1055-1067.
84. Brown, D. M., Zhang, Z., Stephens, E., Dupree, P. & Turner, S. R. (2009) Characterization of IRX10 and IRX10-like reveals an essential role in glucuronoxylan biosynthesis in Arabidopsis. Plant J. 57:732-746.
85. Hermans, C., Porco, S., Verbruggen, N. & Bush, D. R. (2010) Chitinase-like protein CTL1 plays a role in altering root system architecture in response to multiple environmental conditions. Plant Physiol. 152:904-917.
86. Thomson, E., Ferreira-Cerca, S. & Hurt, E. (2013) Eukaryotic ribosome biogenesis at a glance. J. Cell Sci. 126:4815-4821.
87. Shirasaki, D. I. et al. (2012) Network organization of the huntingtin proteomic interactome in mammalian brain. Neuron 75:41-57.
88. Yu, X. et al. (2013) Plastid-localized glutathione reductase2-regulated glutathione redox status is essential for Arabidopsis root apical meristem maintenance. Plant Cell 25:4451-4468.
89. Uhrig, R. G., Labandera, A. M. & Moorhead, G. B. (2013) Arabidopsis PPP family of serine/threonine protein phosphatases: many targets but few engines. Trends Plant Sci. 18:505-513.

Table S1. Metadata of eight samples and three QC arrangements

Samples	File Name	Run order
QC1	ID38360_04_06069L_4981_052318	1
Htt_{ex1}Q21-1	ID38304_01_06069L_4981_052318	2
Htt_{ex1}Q63-2	ID38309_01_06069L_4981_052318	3
Htt_{ex1}Q21-3	ID38306_01_06069L_4981_052318	4
Htt_{ex1}Q63-3	ID38310_01_06069L_4981_052318	5
QC2	ID38360_05_06069L_4981_052318	6
Htt_{ex1}Q63-1	ID38308_01_06069L_4981_052318	7
Htt_{ex1}Q21-2	ID38305_01_06069L_4981_052318	8
Htt_{ex1}Q63-4	ID38311_01_06069L_4981_052318	9
Htt_{ex1}Q21-4	ID38307_01_06069L_4981_052318	10
QC3	ID38360_06_06069L_4981_052318	11

Table S7. Peroxidases involved in ROS generation

UniProtKB	Protein description	Log2 fold-change	*p*-value*
A0A1S3YET7_TOBAC	Peroxidase	-1.64	9.4E-04
A0A1S4DNP1_TOBAC	Peroxidase	-1.41	6.3E-05
A0A1S3ZXH0_TOBAC	Peroxidase	-0.81	1.0E-03
A0A1S3XNL8_TOBAC	Peroxidase	2.56	5.4E-05
A0A1S4BNU3_TOBAC	Peroxidase	2.51	5.8E-05
PERN1_TOBAC	Peroxidase N1	2.42	2.2E-05
A0A1S4D2U8_TOBAC	Peroxidase	2.18	5.3E-04
A0A1S4CK25_TOBAC	Peroxidase	2.16	5.6E-06
A0A1S4AW97_TOBAC	Peroxidase	2.12	7.5E-05
A0A1S3Z5I5_TOBAC	L-ascorbate peroxidase 3, peroxisomal-like	1.91	1.3E-02
A0A1S4A7R5_TOBAC	Peroxidase	1.88	2.5E-04
A0A1S4C127_TOBAC	Peroxidase	1.83	3.0E-04
A0A1S3WXW1_TOBAC	Peroxidase	1.80	4.3E-05
Q50LG5_TOBAC	Peroxidase	1.39	1.0E-03
A0A1S3YS19_TOBAC	Peroxidase	1.38	2.0E-03
A0A1S3YKK2_TOBAC	Peroxidase	1.31	7.0E-03
Q40487_TOBAC	Peroxidase	1.27	1.0E-04
A0A1S4BJB5_TOBAC	Peroxidase	1.18	1.3E-04
A0A1S4BEZ8_TOBAC	Peroxidase	1.13	6.8E-04
A0A1S4D627_TOBAC	Peroxidase	1.10	1.0E-03
A0A1S3Z4R4_TOBAC	Peroxidase	1.08	3.6E-05
A0A1S3XNR7_TOBAC	Glutathione peroxidase	1.02	6.2E-05

A0A1S4CKJ4_TOBAC	L-ascorbate peroxidase 3, peroxisomal	1.02	7.5E-06
A0A1S4CXA3_TOBAC	Peroxidase	1.00	4.9E-05
A0A1S3XQB7_TOBAC	Peroxidase	0.88	1.7E-02
A0A1S3XBP1_TOBAC	Peroxidase	0.87	2.0E-03
A0A1S4C0F0_TOBAC	Peroxidase	0.87	6.4E-05
A0A1S4AIS4_TOBAC	Peroxidase	0.75	1.1E-02
A0A1S3YB42_TOBAC	Glutathione peroxidase	0.65	4.8E-04
A0A1S3ZT94_TOBAC	Peroxidase	0.63	8.0E-03

*: t-test with FDR correction

Table S8. Mitochondrial membrane DAPs involved in transport

UniProtKB	Protein description	Log2 fold-change	*p*-value*
A0A1S3ZIV3_TOBAC	Mitochondrial import inner membrane translocase subunit Tim9	-1.51	1.0E-03
A0A1S4CCA3_TOBAC	Mitochondrial import inner membrane translocase subunit Tim13-like	-1.37	3.3E-04
A0A1S4A7X7_TOBAC	Mitochondrial inner membrane protein OXA1-like	-1.31	5.4E-04
A0A1S3YGL2_TOBAC	Probable mitochondrial import inner membrane translocase subunit TIM21	-0.77	8.4E-04
A0A1S4AKN6_TOBAC	Mitochondrial inner membrane protease ATP23	-0.76	2.4E-02
A9CM21_TOBAC	Voltage-dependent anion channel	0.92	6.1E-05
A0A1S4CVQ8_TOBAC	LETM1 and EF-hand domain-containing protein 1, mitochondrial-like	0.86	2.6E-05
A0A1S4CBH2_TOBAC	Mitochondrial outer membrane protein porin 2-like	0.84	1.4E-04
A0A1S3XKP1_TOBAC	Mitochondrial pyruvate carrier	0.83	4.6E-05
A0A1S4B0F0_TOBAC	Mitochondrial pyruvate carrier	0.80	1.2E-04
A0A1S3YFU4_TOBAC	LETM1 and EF-hand domain-containing protein 1, mitochondrial-like	0.77	1.8E-05
A0A1S3ZVC1_TOBAC	Mitochondrial pyruvate carrier	0.76	8.7E-05
A0A1S3XCA7_TOBAC	Mitochondrial uncoupling protein 5-like	0.76	3.0E-03
A0A1S4DJZ0_TOBAC	Mechanosensitive ion channel protein 1, mitochondrial-like isoform X1	0.62	4.0E-03

*: t-test with FDR correction

Table S9. Mitochondrial DAPs involved in electron transfer chain, amino acid metabolism and TCA cycle

UniProtKB	Protein description	Log2 fold-change	*p*-value*
**Electron transfer chain**			
A0A1S4CJ56_TOBAC	Cytochrome c oxidase assembly protein COX11, mitochondrial-like isoform X1	-0.79	1.2E-02
A0A1S4CBE2_TOBAC	Mitochondrial zinc maintenance protein 1, mitochondrial-like	-0.64	8.5E-04
A0A1S4D561_TOBAC	2-Methylacyl-CoA dehydrogenase, mitochondrial-like isoform X1	2.30	7.5E-06
A0A1S3ZHK0_TOBAC	Formate dehydrogenase, mitochondrial	1.92	3.9E-04
A0A1S3Y6A0_TOBAC	Electron transfer flavoprotein-ubiquinone oxidoreductase, mitochondrial	1.34	2.4E-05
A0A1S3XVJ3_TOBAC	Uncharacterized protein At2g27730, mitochondrial-like isoform X1	1.31	3.2E-05
A0A1S3XC77_TOBAC	Electron transfer flavoprotein subunit alpha, mitochondrial-like	1.23	3.0E-05
AOX1_TOBAC	Ubiquinol oxidase 1, mitochondrial	1.15	9.8E-05
A0A1S3ZC77_TOBAC	Electron transfer flavoprotein subunit beta, mitochondrial-like	1.14	3.5E-05
A0A1S3ZUW5_TOBAC	Internal alternative NAD(P)H-ubiquinone oxidoreductase A1, mitochondrial-like	1.04	1.6E-05
A0A1S4DIL6_TOBAC	Uncharacterized protein At2g27730, mitochondrial-like	0.89	6.0E-05
A0A1S3Z2K5_TOBAC	Aldehyde dehydrogenase family 2 member B7, mitochondrial-like	0.85	3.8E-05
A0A1S4CQH8_TOBAC	Ubiquinone biosynthesis monooxygenase COQ6, mitochondrial	0.81	7.1E-05
A0A1S4C6F3_TOBAC	External alternative NAD(P)H-ubiquinone oxidoreductase B1, mitochondrial	0.79	4.7E-04
A0A1S4B9Y3_TOBAC	Uncharacterized protein At2g27730, mitochondrial-like	0.62	4.6E-04
A0A1S3X0W4_TOBAC	Cytochrome c1-2, heme protein, mitochondrial-like	0.61	2.0E-03
A0A1S3ZKM8_TOBAC	Ubiquinone biosynthesis protein COQ4 homolog, mitochondrial	0.60	1.2E-02
**Amino acid metabolism**			
SYE_TOBAC	Glutamate--tRNA ligase, chloroplastic/mitochondrial	-0.67	5.8E-05
A0A1S3XY89_TOBAC	Arginase 1, mitochondrial	2.01	7.5E-06
A0A1S3Z2C6_TOBAC	Methylcrotonoyl-CoA carboxylase beta chain, mitochondrial-like	1.71	2.5E-05

A0A1S3ZD18_TOBAC	Isovaleryl-CoA dehydrogenase, mitochondrial	1.63	7.1E-06
A0A1S3XVN9_TOBAC	Mitochondrial amidoxime reducing component 2-like isoform X1	1.19	2.3E-04
A0A1S3Z2K7_TOBAC	Methylcrotonoyl-CoA carboxylase subunit alpha, mitochondrial-like	1.19	5.6E-05
A0A1S4DR86_TOBAC	Hydroxymethylglutaryl-CoA lyase, mitochondrial-like	1.04	6.0E-05
A0A1S4C601_TOBAC	Alanine--glyoxylate aminotransferase 2 homolog 1, mitochondrial-like	1.03	5.0E-05
A0A1S4A1B1_TOBAC	Threonine--tRNA ligase, chloroplastic/mitochondrial 2	0.93	1.3E-04
A0A1S4A4G8_TOBAC	Ferredoxin-dependent glutamate synthase 1, chloroplastic/mitochondrial-like	0.89	4.5E-04
A0A1S3Y512_TOBAC	Threonine--tRNA ligase, mitochondrial 1-like isoform X2	0.85	1.1E-02
A0A1S3XS90_TOBAC	Bifunctional dethiobiotin synthetase/7,8-diamino-pelargonic acid aminotransferase, mitochondrial-like isoform X1	0.83	5.7E-04
A0A1S4AZA4_TOBAC	Gamma aminobutyrate transaminase 1, mitochondrial isoform X1	0.80	7.6E-04
A0A1S3ZGF8_TOBAC	Delta-1-pyrroline-5-carboxylate dehydrogenase 12A1, mitochondrial-like	0.62	1.5E-04
A0A1S3ZQY8_TOBAC	Bifunctional D-cysteine desulfhydrase/1-aminocyclopropane-1-carboxylate deaminase, mitochondrial	0.61	5.5E-05
**TCA cycle**			
A0A1S4BDU7_TOBAC	Acylpyruvase FAHD1, mitochondrial-like	0.95	7.1E-05
A0A1S4DJT3_TOBAC	Succinate dehydrogenase subunit 5, mitochondrial-like isoform X2	0.64	1.0E-03
A0A1S4D199_TOBAC	Succinate dehydrogenase subunit 6, mitochondrial-like	0.59	2.0E-03

*: t-test with FDR correction

Table S10. DAPs involved in tubular ER network and root hair elongation

UniProtKB	Protein description	Log2 fold-change	*p*-value*
**Reticulons**			
A0A1S3XSN1_TOBAC	Reticulon-like protein	-1.77	5.7E-06
A0A1S3XEV5_TOBAC	Rreticulon-like protein	-1.65	3.5E-05

A0A1S4CBJ7_TOBAC	Reticulon-like protein	-1.36	1.6E-04
**GTPases**			
A0A1S4BSP5_TOBAC	Nuclear/nucleolar GTPase 2	-1.53	1.0E-04
A0A1S4AAV4_TOBAC	LOW QUALITY PROTEIN: GTPase LSG1-2	-1.04	1.1E-04
A0A1S4DQ39_TOBAC	Protein ROOT HAIR DEFECTIVE 3 homolog	-0.82	4.9E-05
A0A1S4BU20_TOBAC	Mitochondrial Rho GTPase	0.70	2.0E-03
**Actin**			
A0A1S3YSA4_TOBAC	Actin-like	-0.65	1.4E-04
A0A1S3ZMD9_TOBAC	Actin	-0.62	1.4E-04
**Actin binding proteins**			
A0A1S4BZF6_TOBAC	Villin-2-like	-1.27	5.3E-04
A0A1S4DQI1_TOBAC	Formin-like protein 18	-0.80	2.9E-04
A0A1S4DAT8_TOBAC	Myosin-6-like	-0.65	5.9E-05
A0A1S4BQ28_TOBAC	Myosin-1-like	-0.59	5.5E-04
**Phospholipids**			
A0A1S4C3I1_TOBAC	Phosphatidylinositol 3,4,5-trisphosphate 3-phosphatase and protein-tyrosine-phosphatase PTEN2A-like	-1.32	1.2E-04
A0A1S4CNE1_TOBAC	Phosphatidylinositol 4-kinase alpha 1	-0.67	2.0E-03
**Microtubule severing proteins**			
A0A1S4B058_TOBAC	Katanin p60 ATPase-containing subunit A-like 2 isoform X1	-1.89	2.2E-04
A0A1S4BXG6_TOBAC	Katanin p60 ATPase-containing subunit A1	-1.03	2.0E-03
**Calcium binding/dependent proteins**			
Q93YF4_TOBAC	Calcium-dependent protein kinase 2	-1.27	1.5E-05
A0A1S4CK71_TOBAC	Calcium load-activated calcium channel	-1.05	9.6E-05
A0A1S4C2K7_TOBAC	Calcium-transporting ATPase	-1.00	5.9E-05
A0A1S4BDN1_TOBAC	Calcium-transporting ATPase 4, endoplasmic reticulum-type-like	-0.82	2.8E-05
A0A1S3ZRG7_TOBAC	Calcium-dependent protein kinase 5	-0.78	2.9E-04
A0A1S3XR50_TOBAC	Probable calcium-binding protein CML13	-0.76	4.9E-04
A0A1S3X4F2_TOBAC	Calcium-dependent protein kinase 4 isoform X2	-0.70	8.2E-05
A0A1S4BCT9_TOBAC	Calcium-dependent protein kinase 2-like	-0.69	1.9E-05
A0A1S4AQV9_TOBAC	Probable calcium-binding protein CML14	-0.62	4.6E-04
A0A1S3YRP4_TOBAC	Probable calcium-binding protein CML49	1.82	5.1E-05
A0A1S4DAE0_TOBAC	Probable calcium-binding protein CML50	0.88	6.0E-04
**NADPH oxidase**			
Q8RVJ9_TOBAC	NADPH oxidase	-0.60	6.5E-04
**Proton-ATPases**			
A0A1S3YUM1_TOBAC	V-type proton ATPase subunit a	-1.00	1.1E-04

VATG2_TOBAC	V-type proton ATPase subunit G 2	-0.96	1.3E-02
**Vesicle-mediated transporters**			
A0A1S3XFQ2_TOBAC	Exocyst complex component EXO70A1-like	-0.87	7.5E-05
A0A1S3XHT6_TOBAC	Protein transport protein sec16	-1.00	2.8E-05
A0A1S3YKI1_TOBAC	Protein transport protein SEC23-like	-0.65	1.9E-04
A0A1S3YTI3_TOBAC	MAG2-interacting protein 2-like	-0.76	2.0E-03
A0A1S3ZJ13_TOBAC	Clathrin light chain	-0.69	3.6E-05
A0A1S3ZN00_TOBAC	Protein transport protein SEC23-like	-0.93	2.2E-04
A0A1S3ZVR0_TOBAC	Rhomboid-like protein 19	-1.16	3.4E-04
A0A1S4A1T2_TOBAC	Protein transport protein SEC13 homolog B-like	-0.75	4.9E-04
A0A1S4AU43_TOBAC	25.3 kDa vesicle transport protein-like isoform X1	-0.73	5.8E-05
A0A1S4B101_TOBAC	TBC1 domain family member 2B-like isoform X1	-0.70	2.1E-04
A0A1S4B103_TOBAC	Auxilin-related protein 2-like	-0.60	9.0E-03
A0A1S4B7H0_TOBAC	25.3 kDa vesicle transport protein	-0.90	1.2E-04
A0A1S4BHU2_TOBAC	Protein transport protein SEC24-like	-0.65	3.8E-04
A0A1S4BS72_TOBAC	SEC1 family transport protein SLY1-like	-0.63	1.8E-04
A0A1S4CVD2_TOBAC	Golgi SNAP receptor complex member 1	-1.13	2.7E-04
A0A1S4D9U5_TOBAC	Protein transport protein SEC23-like	-0.89	5.2E-04
A0A1S4DH90_TOBAC	Coatomer subunit beta'-1-like	-0.64	8.6E-05
A0A1S4DRM1_TOBAC	Protein transport protein Sec24-like At3g07100	-0.76	4.3E-05
Q9SDQ5_TOBAC	GTP-binding protein SAR1A-like	-0.67	4.3E-05

*: t-test with FDR correction

Table S11. DAPs involved in ribosome biogenesis and ribosome assembly

UniProtKB	Protein description	Log2 fold-change	*p*-value*
**Ribosome biogenesis related proteins**			
A0A1S3Y752_TOBAC	Ribosome biogenesis protein NSA2 homolog	-1.59	2.5E-05
A0A1S3ZMA0_TOBAC	Ribosome biogenesis protein BMS1 homolog	-1.31	1.4E-04
A0A1S3Z5Y9_TOBAC	Ribosome biogenesis regulatory protein	-1.28	8.1E-05
A0A1S4BK64_TOBAC	Ribosome biogenesis protein WDR12 homolog	-1.25	5.5E-05

A0A1S3XGV3_TOBAC	Putative ribosome biogenesis protein C8F11.04	-1.12	2.8E-05
A0A1S3Z0I4_TOBAC	Ribosome biogenesis protein BOP1 homolog	-1.08	6.4E-05
A0A1S4DAU9_TOBAC	Ribosome biogenesis protein BRX1 homolog	-0.76	3.7E-04
**AAA-type ATPases**			
A0A1S4BN34_TOBAC	ATPase family AAA domain-containing protein 1-like	-1.26	1.0E-03
A0A1S4CWQ5_TOBAC	AAA-ATPase ASD, mitochondrial-like	0.92	3.0E-03
A0A1S4BZ22_TOBAC	AAA-ATPase At3g28580-like	0.72	9.0E-03
**Helicases**			
A0A1S4DI29_TOBAC	DEAD-box ATP-dependent RNA helicase 10-like	-1.60	5.5E-05
A0A1S3XE50_TOBAC	ATP-dependent RNA helicase-like protein DB10 isoform X1	-1.49	1.0E-03
A0A1S4A4Z1_TOBAC	DEAD-box ATP-dependent RNA helicase 9-like isoform X1	-1.48	2.4E-05
A0A1S4C7R7_TOBAC	DEAD-box ATP-dependent RNA helicase 7-like	-1.43	8.9E-05
A0A1S3ZEG5_TOBAC	DEAD-box ATP-dependent RNA helicase 53-like isoform X1	-1.40	3.4E-05
A0A1S3YXI7_TOBAC	DEAD-box ATP-dependent RNA helicase 31-like	-1.33	1.6E-04
A0A1S3YSR9_TOBAC	Putative DEAD-box ATP-dependent RNA helicase 29	-1.31	6.0E-05
A0A1S3XY80_TOBAC	ATP-dependent RNA helicase-like protein DB10 isoform X1	-1.26	5.3E-05
A0A1S4A617_TOBAC	ATP-dependent RNA helicase A-like protein	-1.22	1.2E-04
A0A1S3XMH8_TOBAC	LOW QUALITY PROTEIN: DExH-box ATP-dependent RNA helicase DExH9-like	-1.12	1.4E-02
A0A1S3YK03_TOBAC	DEAD-box ATP-dependent RNA helicase 13-like	-1.10	9.5E-04
A0A1S3X981_TOBAC	DEAD-box ATP-dependent RNA helicase 57 isoform X1	-0.97	4.1E-04
A0A1S3YVT9_TOBAC	DEAD-box ATP-dependent RNA helicase 16-like	-0.77	3.0E-03
A0A1S3ZS83_TOBAC	DEAD-box ATP-dependent RNA helicase 5-like	-0.72	1.6E-04
**GTPases**			
A0A1S4BSP5_TOBAC	Nuclear/nucleolar GTPase 2	-1.53	1.0E-04
A0A1S3X678_TOBAC	Probable ADP-ribosylation factor GTPase-activating protein AGD6	-1.41	5.9E-04

A0A1S4AAV4_TOBAC	LOW QUALITY PROTEIN: GTPase LSG1-2	-1.04	1.1E-04
A0A1S4BU20_TOBAC	Mitochondrial Rho GTPase	0.70	2.0E-03

*: t-test with FDR correction

Table S12. DAPs involved in regulation of glutathione redox status

UniProtKB	Protein description	Log2 fold-change	*p*-value*
**Glutathione *S*-transferases**			
A0A1S3Y307_TOBAC	Glutathione S-transferase U10-like	2.49	1.0E-03
GSTX4_TOBAC	Probable glutathione S-transferase	2.47	2.1E-04
GSTX4_TOBAC	Probable glutathione S-transferase	2.47	2.1E-04
GSTX3_TOBAC	Probable glutathione S-transferase	2.37	1.0E-03
GSTXA_TOBAC	Probable glutathione S-transferase parA	2.36	8.5E-04
A0A1S4CQJ4_TOBAC	Glutathione S-transferase U9-like	2.30	1.0E-03
A0A1S4AJB2_TOBAC	Probable glutathione S-transferase	2.23	1.0E-03
A0A1S3YEE6_TOBAC	Glutathione S-transferase U9-like	2.08	6.1E-04
A0A1S3YUL8_TOBAC	Glutathione S-transferase-like	2.02	9.1E-05
A0A1S3X0J2_TOBAC	Glutathione S-transferase L3-like isoform X1	1.85	4.3E-05
A0A1S4BDE5_TOBAC	Probable glutathione S-transferase	1.76	1.0E-03
GSTX1_TOBAC	Probable glutathione S-transferase	1.73	5.3E-04
GSTF2_TOBAC	Glutathione S-transferase APIC	1.71	3.5E-04
A0A1S4BZT5_TOBAC	Probable glutathione S-transferase	1.70	1.0E-03
Q40533_TOBAC	ParB product which have an activity of glutathione S-transferase	1.66	5.8E-04
A0A1S4AX14_TOBAC	Probable glutathione S-transferase	1.64	1.0E-04
A0A1S3YM66_TOBAC	Probable glutathione S-transferase parC	1.64	3.0E-03
A0A1S4BT19_TOBAC	Glutathione S-transferase zeta class-like	1.55	2.0E-03
A0A1S4BFF8_TOBAC	Probable glutathione S-transferase	1.40	2.7E-04
A0A1S3Z4V2_TOBAC	Glutathione S-transferase U8-like	1.34	3.0E-03
A0A1S4CJX9_TOBAC	Glutathione S-transferase T1-like	1.32	1.0E-03
A0A1S4CS55_TOBAC	Glutathione transferase GST 23-like	1.31	3.0E-03
A0A1S3XIX1_TOBAC	Probable glutathione S-transferase	1.24	7.6E-04
A0A1S3Y8V8_TOBAC	Glutathione S-transferase DHAR3, chloroplastic	1.18	9.0E-03
A0A1S4DMX8_TOBAC	Probable glutathione S-transferase	0.88	9.0E-03
A0A1S4A868_TOBAC	Glutathione S-transferase	0.87	3.0E-03
A0A1S4A502_TOBAC	Glutathione S-transferase T1-like	0.85	3.9E-05

A0A1S3YQ00_TOBAC	Microsomal glutathione S-transferase 3-like	0.73	4.2E-04
**Other glutathione metabolism related proteins**			
A0A1S3YKT8_TOBAC	Glutathione reductase, chloroplastic-like isoform X1	1.47	2.0E-04
A0A1S4B721_TOBAC	Lactoylglutathione lyase	1.06	4.1E-06
A0A1S4AJD1_TOBAC	Probable lactoylglutathione lyase, chloroplastic isoform X1	0.90	6.0E-05
A0A077D837_TOBAC	Glutathione synthetase	0.71	8.8E-04
A0A1S3XER8_TOBAC	S-formylglutathione hydrolase	0.67	9.8E-05
A0A1S3YB42_TOBAC	Glutathione peroxidase	0.65	4.8E-04
GSHRP_TOBAC	Glutathione reductase, chloroplastic	0.62	7.6E-05
A0A1S3XF65_TOBAC	Lactoylglutathione lyase	0.61	1.4E-04

*: t-test with FDR correction

Table S13. RNA polymerases involved in transcription

UniProtKB	Protein description	Log2 fold-change	*p*-value*
A0A1S4BU78_TOBAC	RNA polymerase II-associated protein 3-like	-1.11	1.4E-04
A0A1S4CS69_TOBAC	DNA-directed RNA polymerases IV and V subunit 4-like	-0.73	5.0E-04
A0A1S3XKG9_TOBAC	DNA-directed RNA polymerase I subunit rpa49-like isoform X1	-0.64	1.0E-03
A0A1S4DES6_TOBAC	DNA-directed RNA polymerases I and III subunit RPAC2-like isoform X1	-0.62	2.5E-04
A0A1S4BBD8_TOBAC	DNA-directed RNA polymerase I subunit 1-like	-0.61	2.0E-03
A0A1S3YG40_TOBAC	DNA-directed RNA polymerases I and III subunit rpac1-like isoform X2	-0.60	1.6E-04

*: t-test with FDR correction

Table S14. Serine/threonine-protein kinases and phosphatases involved in cell proliferation and expansion

UniProtKB	Protein Description	Log2 fold-change	*p*-value*
A0A1S4A212_TOBAC	Serine/threonine-protein kinase 38-like	-1.72	1.0E-03

A0A1S4AK92_TOBAC	Serine/threonine-protein kinase SAPK7-like	-1.39	5.6E-05
A0A1S3YRK2_TOBAC	Probable LRR receptor-like serine/threonine-protein kinase At1g05700	-1.33	4.6E-04
A0A1S3ZMS6_TOBAC	Serine/threonine-protein kinase 16-like	-1.25	4.9E-04
A0A1S4BJC6_TOBAC	Serine/threonine-protein kinase RUNKEL	-1.11	1.4E-02
A0A1S3YND7_TOBAC	Probable LRR receptor-like serine/threonine-protein kinase At4g20940 isoform X1	-1.08	1.0E-03
A0A1S4DKM3_TOBAC	Serine/threonine-protein kinase HT1-like	-1.01	2.0E-03
G5ELZ4_TOBAC	Serine/threonine-protein phosphatase 5	-0.83	1.1E-04
A0A1S3ZN95_TOBAC	Putative leucine-rich repeat receptor-like serine/threonine-protein kinase At2g14440	-0.82	3.2E-04
A0A1S3ZLP5_TOBAC	Probable serine/threonine-protein kinase DDB_G0280111	-0.81	2.0E-03
A0A1S4D0Z6_TOBAC	Serine/threonine-protein kinase EDR1-like	0.76	2.0E-03

*: t-test with FDR correction

Table S15. DAPs involved in folate and one carbon metabolisms

UniProtKB	Protein description	Log2 fold-change	*p*-value*
**Folate and one carbon metabolism**			
A0A1S3WYB9_TOBAC	GTP cyclohydrolase 1	-2.64	2.0E-03
A0A1S3Y8H2_TOBAC	Probable methyltransferase PMT8	-1.70	3.0E-03
A0A1S4AR74_TOBAC	Probable methyltransferase PMT20	-1.70	3.1E-04
A0A1S4B3D7_TOBAC	Uncharacterized protein At3g49720-like	-1.64	1.1E-04
A0A1S4BH87_TOBAC	Probable methyltransferase PMT5 isoform X1	-1.61	3.3E-05
A0A1S3XT44_TOBAC	rRNA adenine N(6)-methyltransferase	-1.59	4.1E-04
A0A1S3YJW9_TOBAC	Histone-lysine N-methyltransferase, H3 lysine-9 specific SUVH4-like	-1.41	4.1E-04
A0A1S3ZLI4_TOBAC	Probable methyltransferase PMT13	-1.41	5.4E-05
A0A1S4BNH6_TOBAC	Probable methyltransferase PMT2	-1.40	1.3E-04
A0A1S3Y8F0_TOBAC	Probable methyltransferase PMT1	-1.37	7.8E-05
A0A1S3WXP6_TOBAC	Probable methyltransferase PMT3	-1.35	2.2E-04
A0A1S3ZFC4_TOBAC	Probable methyltransferase PMT26	-1.33	1.7E-04
W8TFR1_TOBAC	GTP cyclohydrolase II	-1.32	2.5E-05
A0A1S4D663_TOBAC	Probable methyltransferase PMT14	-1.32	4.2E-05
A0A1S4CPT8_TOBAC	Probable 18S rRNA (Guanine-N(7))-methyltransferase	-1.17	5.9E-04
A0A1S3XBX4_TOBAC	Phosphoethanolamine N-methyltransferase 1-like	-1.16	3.1E-05

A0A1S4A3W8_TOBAC	(RS)-norcoclaurine 6-O-methyltransferase-like	-1.05	5.0E-03
A0A1S3XLU7_TOBAC	tRNA (Cytosine(34)-C(5))-methyltransferase-like	-1.04	4.6E-04
A0A1S3X806_TOBAC	Putative rRNA methyltransferase	-1.01	2.4E-04
A0A1S3ZBS6_TOBAC	Methyltransferase-like protein 13	-0.96	8.1E-05
A0A1S4CZU1_TOBAC	Probable protein arginine N-methyltransferase 1	-0.94	3.8E-05
A0A1S4BY02_TOBAC	Probable protein arginine N-methyltransferase 3	-0.90	3.5E-04
A0A1S4B9Y5_TOBAC	Alpha N-terminal protein methyltransferase 1-like isoform X1	-0.89	4.2E-04
A0A1S3Y6U4_TOBAC	Glucuronoxylan 4-O-methyltransferase 3-like	-0.89	5.1E-04
A0A1S4CCM9_TOBAC	Protein arginine N-methyltransferase	-0.88	4.8E-05
A0A1S3Y1B2_TOBAC	Probable pectin methyltransferase QUA2	-0.86	7.7E-04
A0A1S3XBQ8_TOBAC	Probable 28S rRNA (Cytosine-C(5))-methyltransferase	-0.82	4.6E-04
A0A1S3X4I9_TOBAC	2-Methyl-6-phytyl-1,4-hydroquinone methyltransferase, chloroplastic-like	-0.75	2.5E-05
A0A1S4CW49_TOBAC	Probable methyltransferase PMT15	-0.75	1.7E-04
CAMT2_TOBAC	Caffeoyl-CoA O-methyltransferase 2	-0.74	4.0E-03
A0A1S4ASH5_TOBAC	Protein arginine N-methyltransferase PRMT10-like	-0.73	2.7E-04
A0A1S3ZEV6_TOBAC	tRNA (Guanine(10)-N2)-methyltransferase homolog	-0.69	3.0E-03
A0A1S4C1X0_TOBAC	tRNA (guanine-N(7)-)-methyltransferase	-0.65	5.5E-04
A0A1S4DN28_TOBAC	DNA (Cytosine-5)-methyltransferase DRM2-like isoform X1	-0.64	3.0E-03
A0A1S3X4H4_TOBAC	tRNA (guanine(26)-N(2))-dimethyltransferase	-0.63	3.9E-04
A0A1S3YCG8_TOBAC	Putative methyltransferase NSUN6 isoform X2	-0.61	2.0E-03
A0A1S4B0J2_TOBAC	Probable methyltransferase PMT11	-0.60	3.0E-03
PMT4_TOBAC	Putrescine *N*-methyltransferase 4	-0.60	1.5E-02
A0A1S3YAK7_TOBAC	Probable methyltransferase PMT28	-0.59	5.0E-03
A0A1S3YGI7_TOBAC	Putative methyltransferase C9orf114 isoform X1	-0.59	1.9E-04
A0A1S4D868_TOBAC	Putative methyltransferase DDB_G0268948	3.02	9.9E-05
A0A1S4A958_TOBAC	Probable caffeoyl-CoA O-methyltransferase At4g26220	1.01	1.0E-03
A0A1S3YDV3_TOBAC	Methionine S-methyltransferase	0.73	1.3E-05

*: t-test with FDR correction

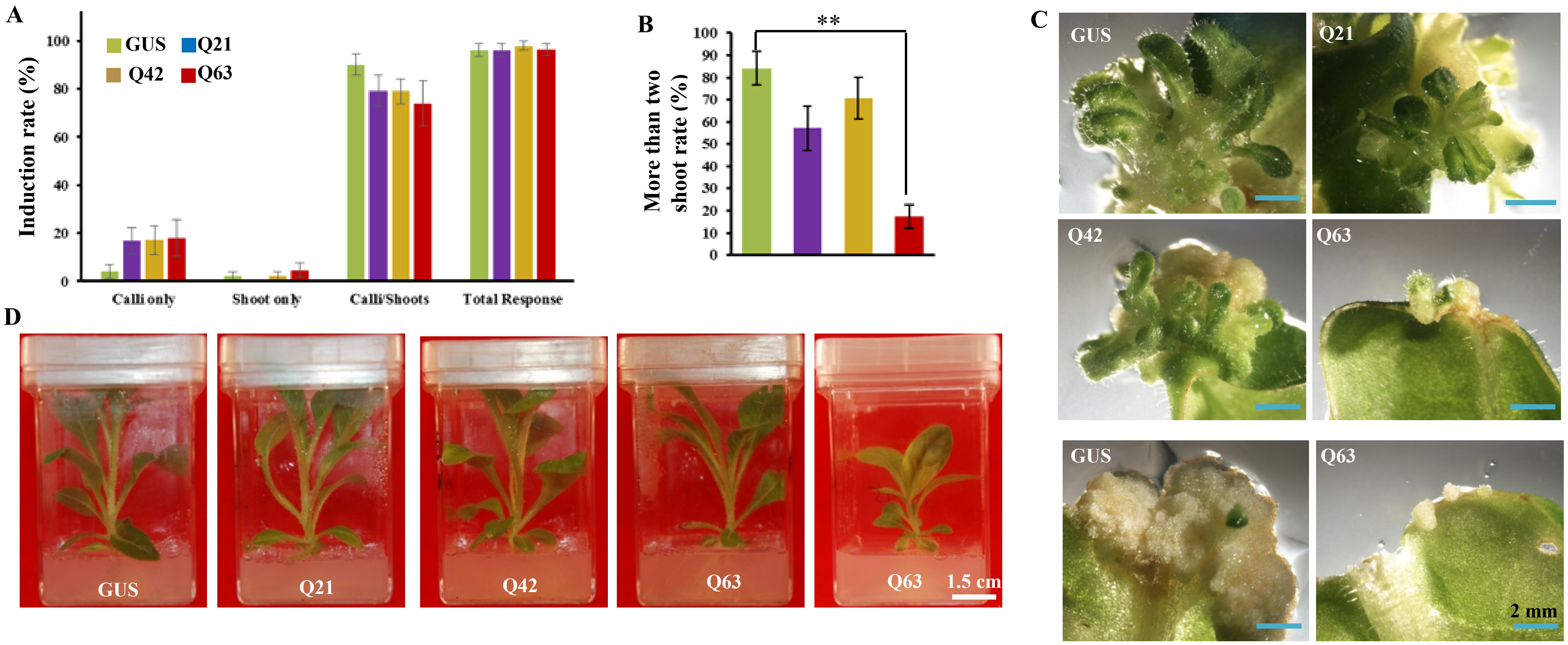

Fig. S1. Transformation efficiency and regenerated transgenic plants. (A) Culture response was counted as the percentage of regenerated calli, shoots, or both from each explant at week four of post-inoculation onto kanamycin selection medium. The average frequency of four plates (five explants per plate) from each genetic cassette per experiment was calculated. Data plotted was the average of three independent experiments ± SD. **(B)** The frequency of explants bearing more than two shoots per shoot formation site was calculated. Data plotted was the average of three independent experiments ± SD. **: significant differences at p <0.01 level. **(C)** Induced shoots from four genetic cassettes, and induced calli from GUS compared to those from $Htt_{ex1}Q63$. **(D)** Representatives of kanamycin-resistant plants from four genetic cassettes were grown on maintenance medium under ~60 µmol m^{-2} s^{-1} light intensity. Plants carrying GUS, $Htt_{ex1}Q21$, $Htt_{ex1}Q42$ grew normally while plants carrying $Htt_{ex1}Q63$ showed some growth defects. Q21: $Htt_{ex1}Q21$; Q42: $Htt_{ex1}Q42$; and Q63: $Htt_{ex1}Q63$.

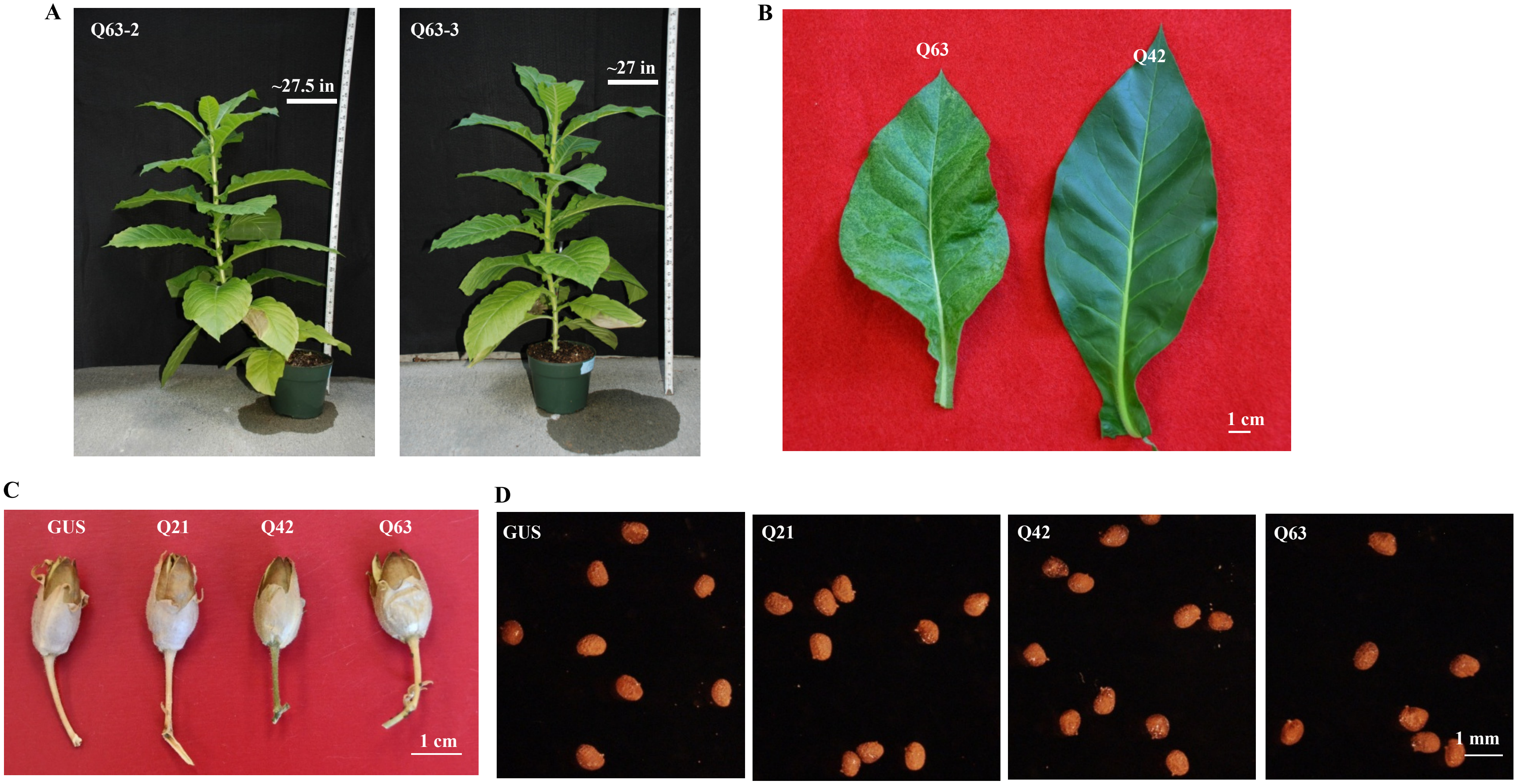

Fig. S2. Transgenic plants, leaves, seed pods and seeds. (A) $Htt_{ex1}Q63$ transgenic plants grew in soil under conditions of ~15 µmol m^{-2} s^{-1} photo-period (16 h d^{-1}) at 23ºC. After eight weeks, median plant height reached approximately 20 to 30 inches (in). **(B)** Representatives of young leaves from soil grown $Htt_{ex1}Q63$ and $Htt_{ex1}Q42$ transgenic plants under conditions of ~15 µmol m^{-2} s^{-1} photo-period (16 h d^{-1}) at 23ºC. Color defective leaf was observed only from $Htt_{ex1}Q63$ but not $Htt_{ex1}Q42$ plants. **(C)** Seed pods of GUS, $Htt_{ex1}Q21$, $Htt_{ex1}Q42$ and $Htt_{ex1}Q63$ transgenic lines were similar in sizes. **(D)** Seeds from the four transgenic seed pods shown in C were also similar in size and shape. Q21: $Htt_{ex1}Q21$; Q42: $Htt_{ex1}Q42$; and Q63: $Htt_{ex1}Q63$.

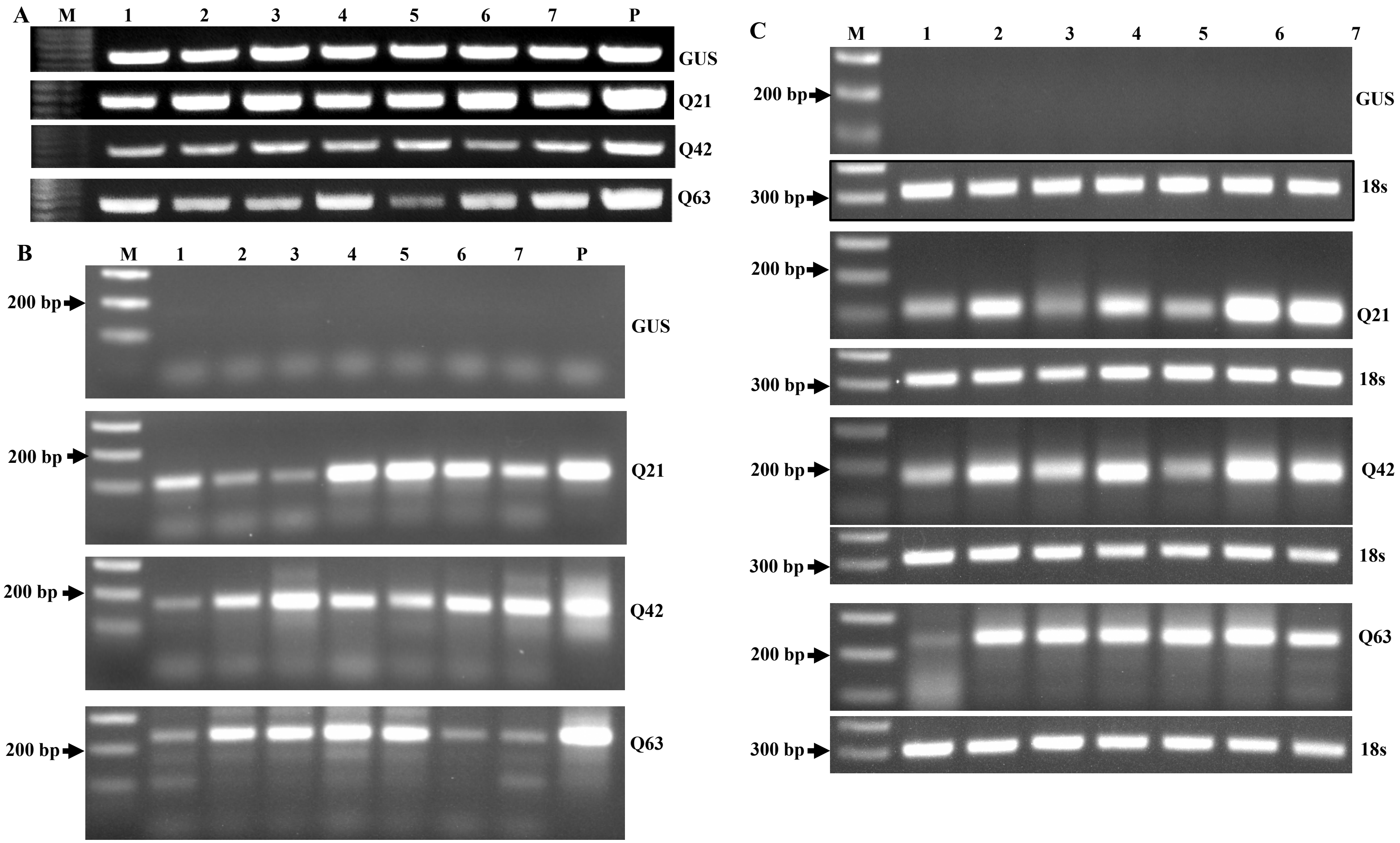

Fig. S3. PCR and RT-PCR results of seven transgenic lines per genetic cassette. (A) Genomic PCR of *nptII* performed with primers NPT-II 5'/NPT-II 3'. All had 795 bp PCR products. **(B)** Genomic PCR of *Htt* exon 1 with different "CAG" repeats performed with primers HDJamaF/HDJamaR2. $Htt_{ex1}Q21$, $Htt_{ex1}Q42$ and $Htt_{ex1}Q63$ transgenic lines had ~110 bp, ~170 bp and ~240 bp PCR products, respectively. GUS lines were negative of PCR. **(C)** RT-PCR of *Htt* exon 1 transcripts with different "CAG" repeats performed using primers HDJamaF/HDJamaR2 whose amplification covered the "CAG" repeat region. *18S* rRNA was used to show equal cDNA used for RT-PCR. $Htt_{ex1}Q21$, $Htt_{ex1}Q42$ and $Htt_{ex1}Q63$ transgenic lines had ~110 bp, ~170 bp and ~240 bp PCR products, respectively. GUS transgenic lines were negative without PCR products. P: Plasmid DNA. M: DNA ladder. Q21: $Htt_{ex1}Q21$; Q42: $Htt_{ex1}Q42$; and Q63: $Htt_{ex1}Q63$.

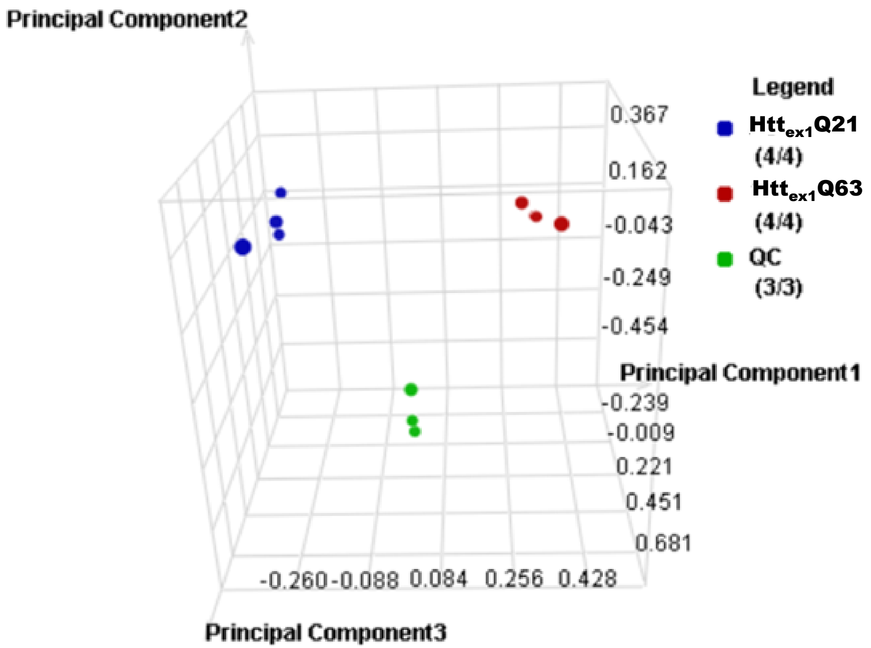

Fig. S4. Principal component analysis of quantified proteins. The expression values of the 5,073 proteins quantified by 2 or more peptides were Z-score-normalized followed by PCA using Rosetta Elucidator.

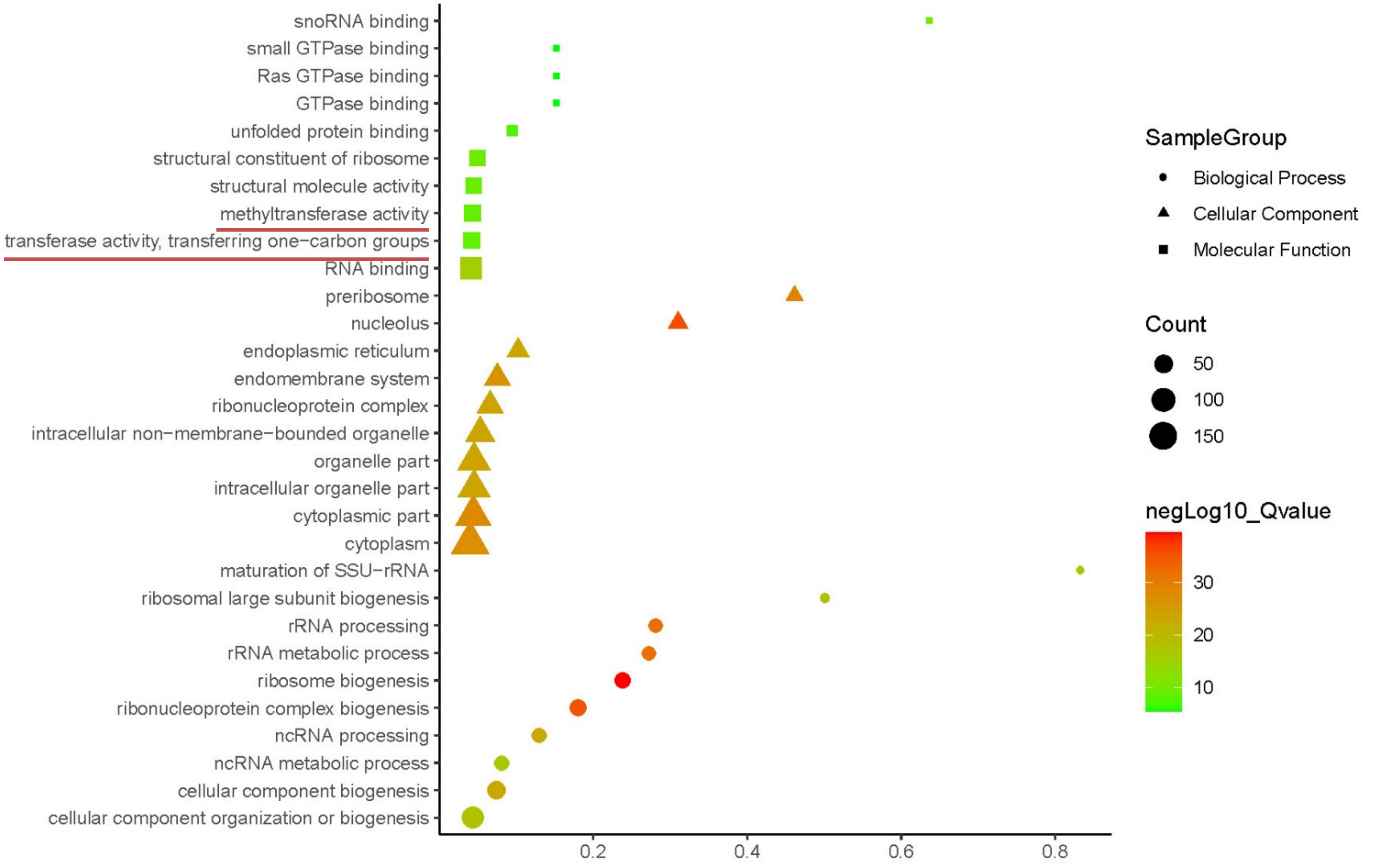

Fig. S5. Selected top 10 GO results of abundance-decreased DAPs.

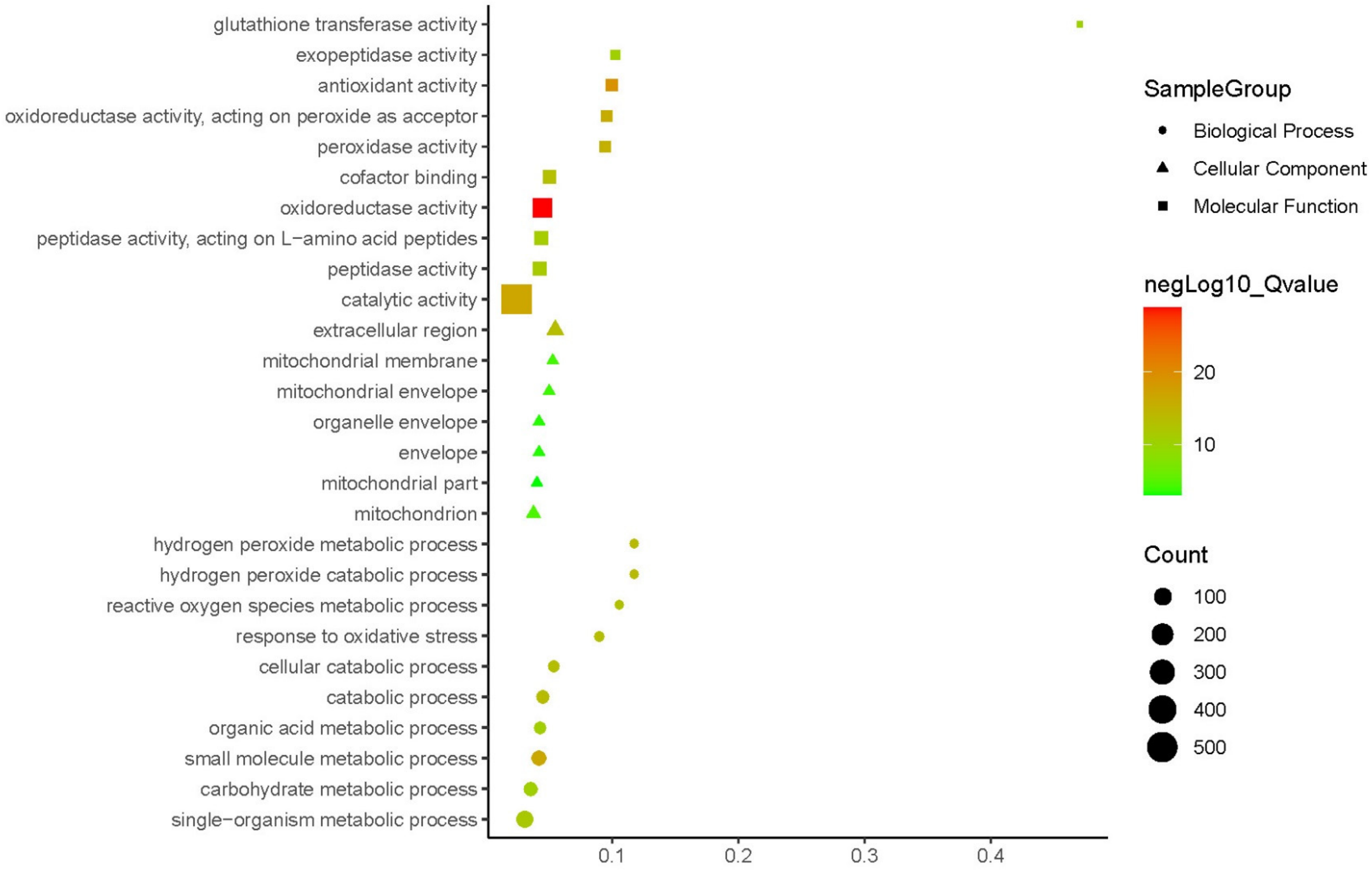

Fig. S6. Selected top 10 GO results of GO results of abundance-increased DAPs. For cellular component category, there are only 7 GO terms.

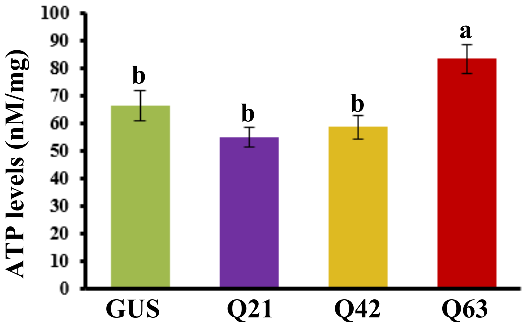

Fig. S7. ATP levels of young leaf tissues. ATP levels of young leaf tissues from four types of transgenic plants. Bioluminescent Assay Kit was used. Data plotted are the average of five biological repeats ± SD (n = 5). Different letters represent significant differences at $p < 0.05$ level.

A

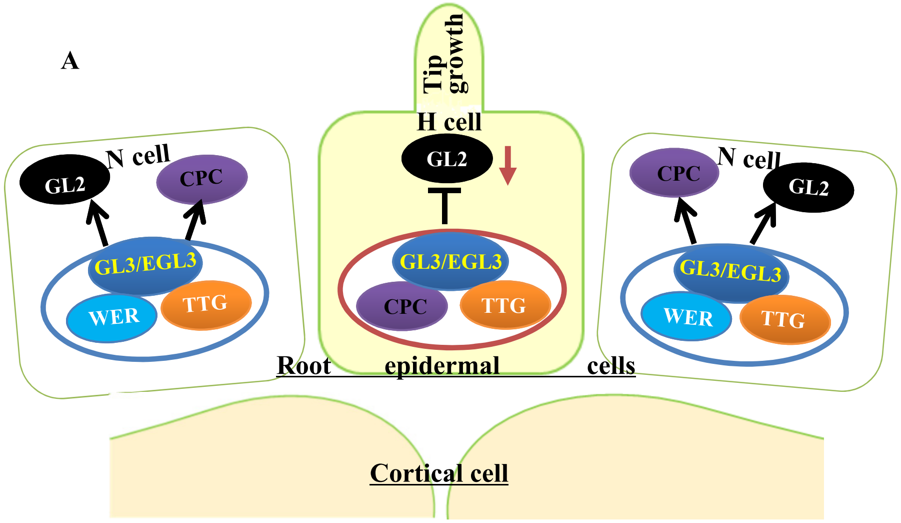

B

GL2 (Q63 vs. Q21 roots): -1.51-fold

C

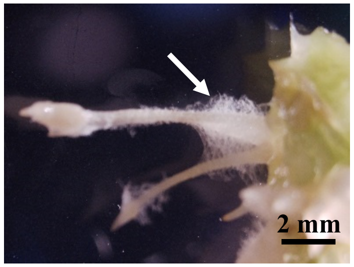

Fig. S8. Root hair initiation in induced roots. (A) Schematic diagram showing the differentiation of epidermal cells with related transcription factors adopted from Libault et al.[30]. The root epidermal cell can be differentiated into a root hair (H) or non-hair (N) cell. The process is determined by a group of transcription factors: GLABRA2 (GL2), CAPRICE (CPC), TRANSPARENT TESTA GLABRA (TTG), GLABRA3 (GL3)/ENHANCER OF GLABRA3 (EGL3) and WEREWOLF (WER). An epidermal cell becomes a H cell when GL2 was inhibited by the formation of CPC-TTG-GL3-EGL3 complex while it becomes a N cell when GL2 was induced by the formation of WER-TTG-GL3-EGL3. The red arrow indicates GL2 abundance was decreased. **(B)** GL2 protein level in Htt_{ex1}Q63 roots was reduced of 1.5-fold compared to Htt_{ex1}Q21 roots. **(C)** Induced roots having hairs when shoots were inoculated on the surface of medium.

A

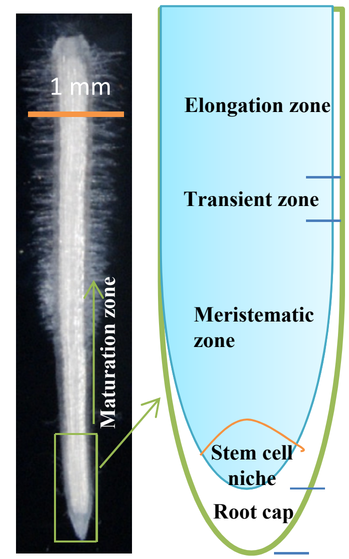

B

Accession #	Protein description	FC*	Function**
Q9AYN9	NQK1 MAPKK	-3.2	Cell division[82]
A0A1S4C0N4	Cyclin-dependent kinases regulatory subunit	-3	Cell division[84]
A0A1S4B058	Katanin p60 ATPase-containing subunit A-like 2 isoform X1	-3.7	Cell elongation[79]
A0A1S4BXG6	Katanin p60 ATPase-containing subunit A1	-2	Cell elongation[79]
Q8GZV0	Obtusifoliol-14-demethylase	-3.4	Cell elongation[81]
A0A1S3X0S9	Probable beta-1,4-xylosyltransferase IRX10L	-2.8	Cell elongation[83]
A0A1S3YHB2	Wall-associated receptor kinase 2-like	-2.3	Cell expansion[80]
A0A1S4CA37	Chitinase-like protein 1	-3.4	Cell expansion[84]
A0A1S3YBC1	Protein ROOT INITIATION DEFECTIVE 3-like	-1.9	Cell expansion[34]

C **I-1: Ribosome biogenesis proteins; I-2: AAA-type ATPases; I-3: Helicases; I-4: GTPases.**
II-1: Glutathione *S*-transferases; II-2: Other glutathione metabolism related proteins.
III: RNA polymerases.
IV: Serine/threonine-protein kinases and phosphatases.

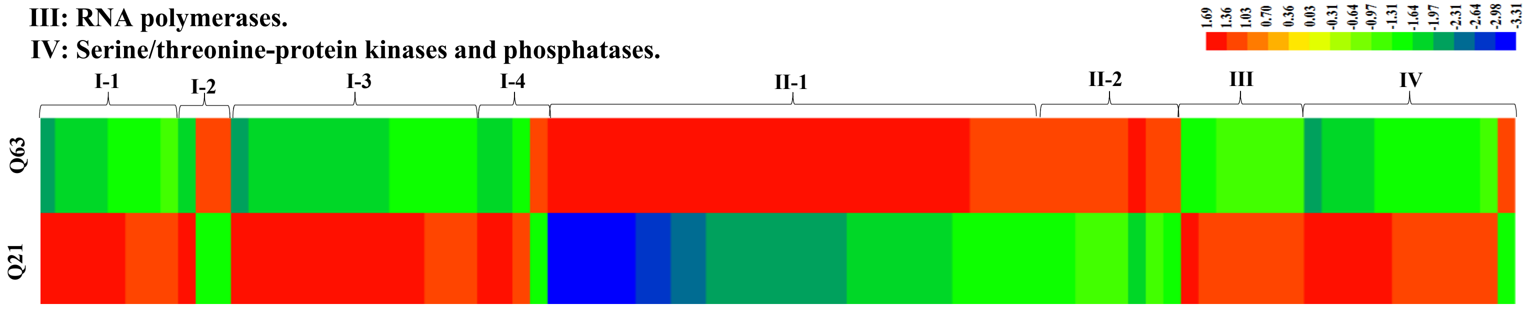

Fig. S9. Normal root tip structure and DAPs associated with root cell division, elongation or expansion. (A) Normal root tip structure. **(B)** DAPs associated with root cell division, elongation or expansion. *: Fold changes of Htt_{ex1}Q63 (Q63) vs. Htt_{ex1}Q21 (Q21); **: the references cited. **(C)** Four groups (I - IV) of DAPs associated with root growth and root cap integrity. Expression values are shown as a color scale at the right corner of the figure.

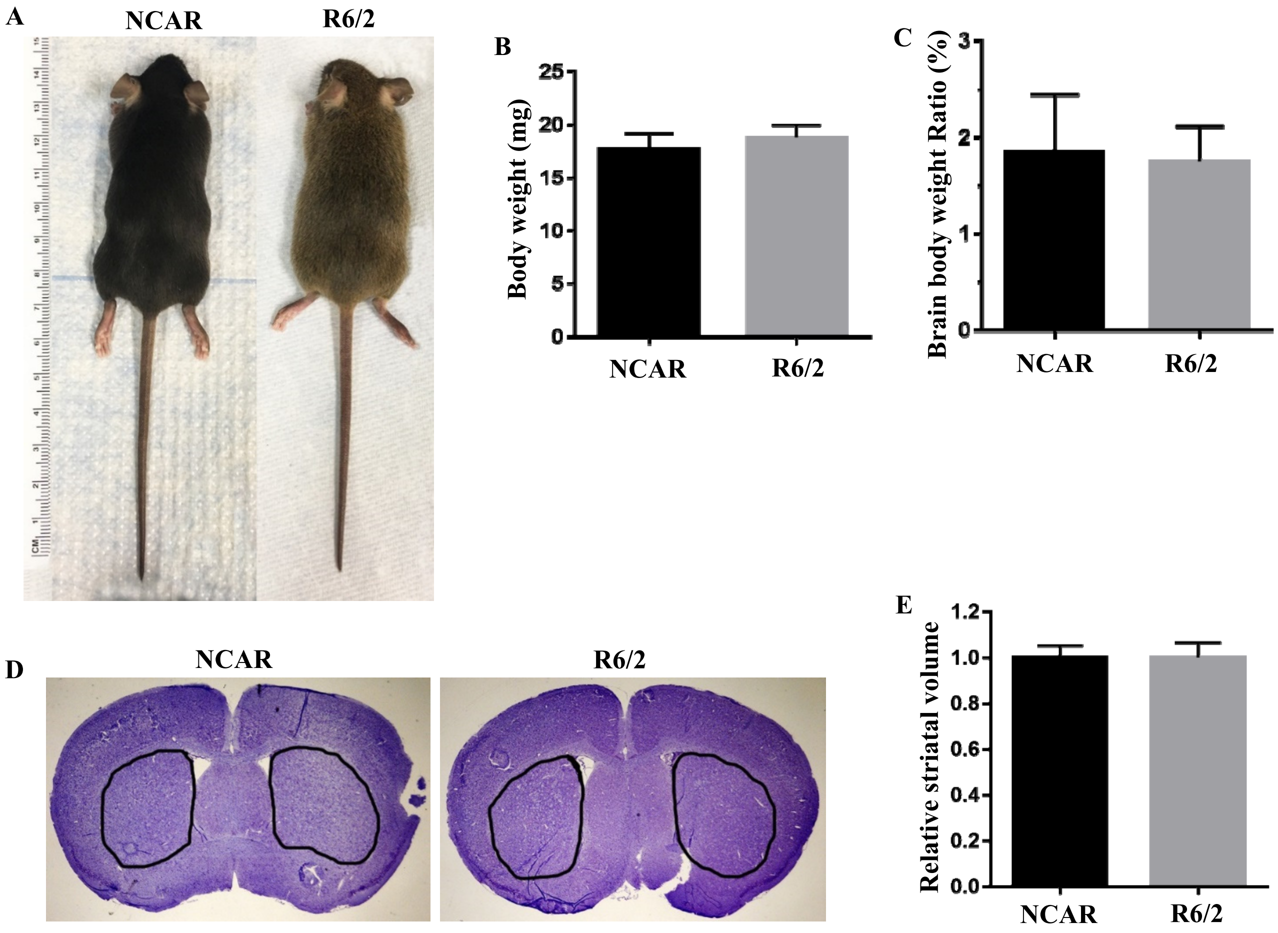

Fig. S10. Characterization of 4-week old wide-type non-carrier (NCAR) and R6/2 HD mice. (A) Mouse phenotype. **(B)** Body weight (n = 7). **(C)** Brain body weight ratio (n = 7). **(D)** Brain section with striatum region circled. **(E)** Relative striatal volume.

Fig. S11

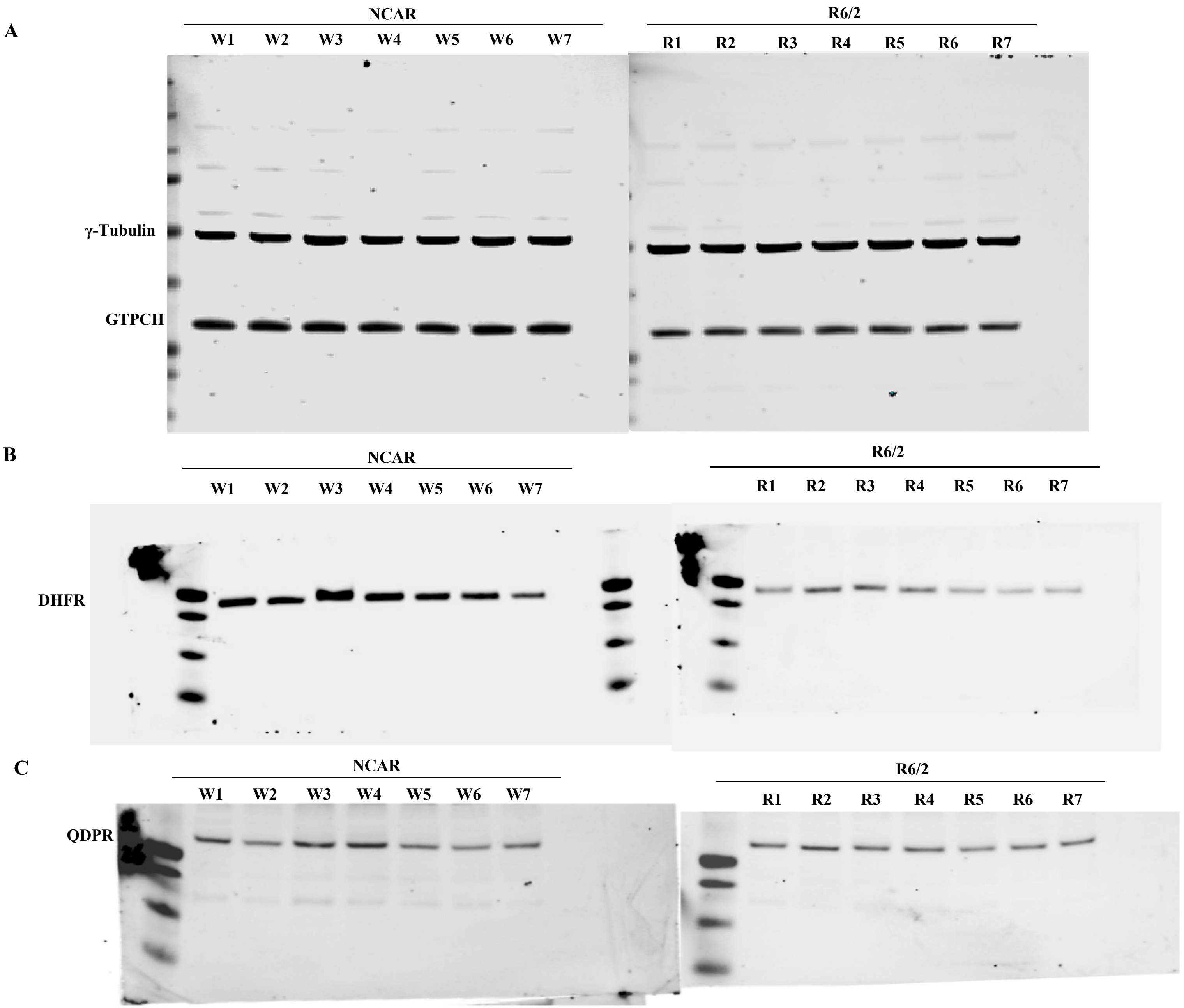
A
NCAR
W1 W2 W3 W4 W5 W6 W7
R6/2
R1 R2 R3 R4 R5 R6 R7
γ-Tubulin
GTPCH
B
NCAR
W1 W2 W3 W4 W5 W6 W7
R6/2
R1 R2 R3 R4 R5 R6 R7
DHFR
C
NCAR
W1 W2 W3 W4 W5 W6 W7
R6/2
R1 R2 R3 R4 R5 R6 R7
QDPR

Fig. S11

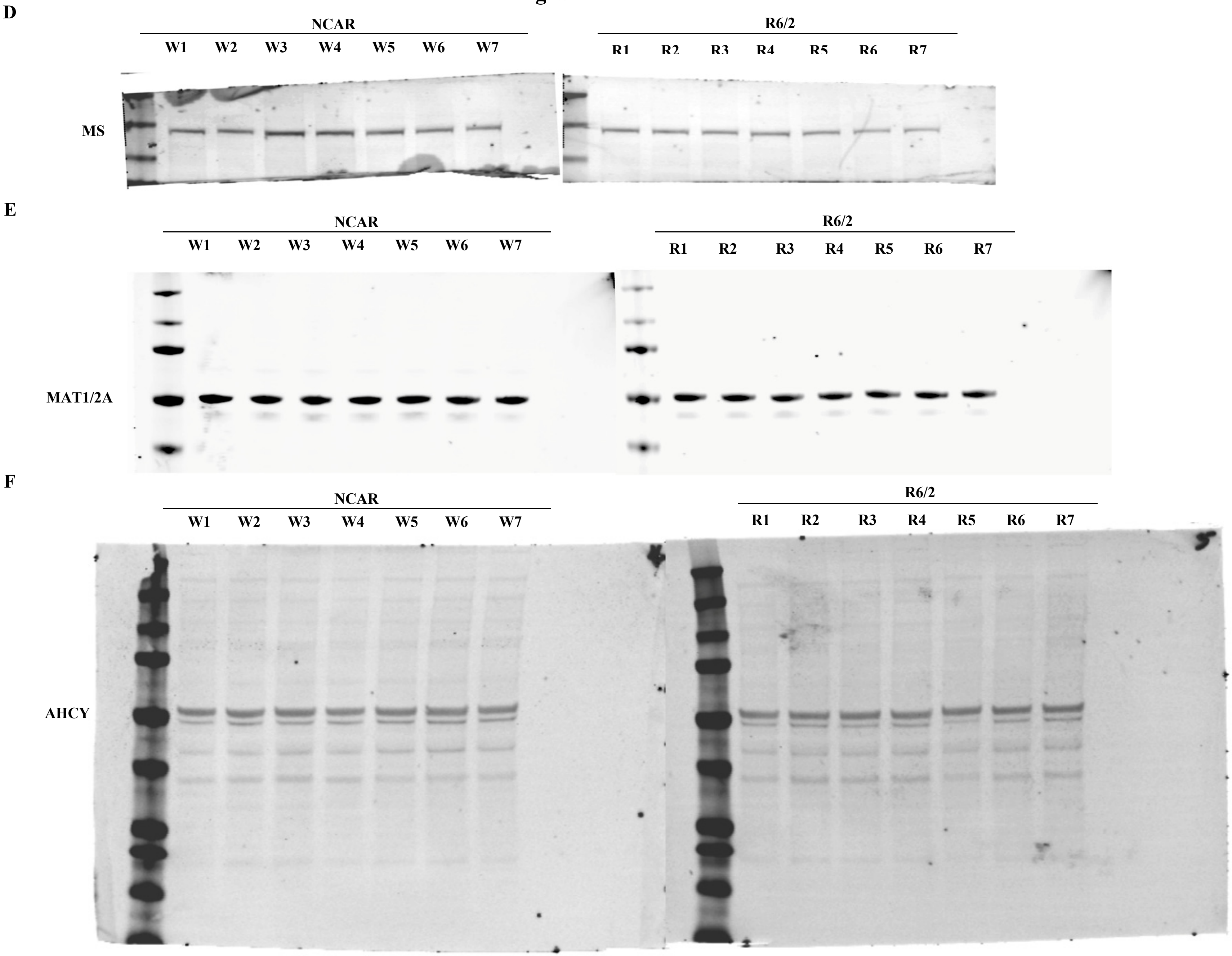
D
NCAR
W1 W2 W3 W4 W5 W6 W7
R6/2
R1 R2 R3 R4 R5 R6 R7
MS
E
NCAR
W1 W2 W3 W4 W5 W6 W7
R6/2
R1 R2 R3 R4 R5 R6 R7
MAT1/2A
F
NCAR
W1 W2 W3 W4 W5 W6 W7
R6/2
R1 R2 R3 R4 R5 R6 R7
AHCY

Fig. S11

G

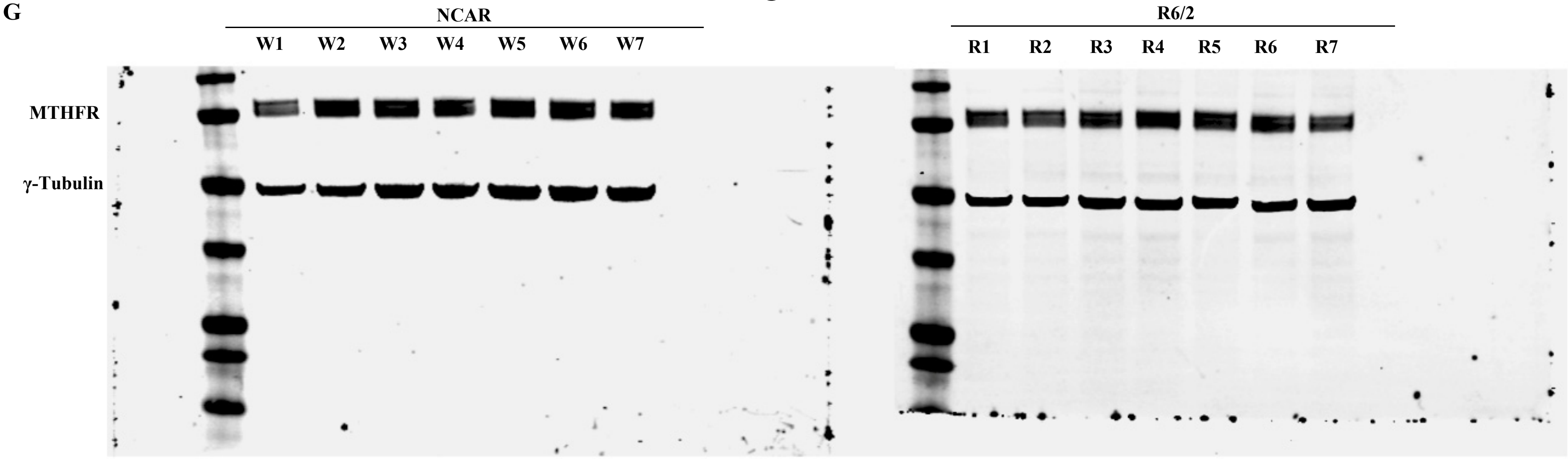
NCAR
W1 W2 W3 W4 W5 W6 W7
R6/2
R1 R2 R3 R4 R5 R6 R7
MTHFR
γ-Tubulin

H

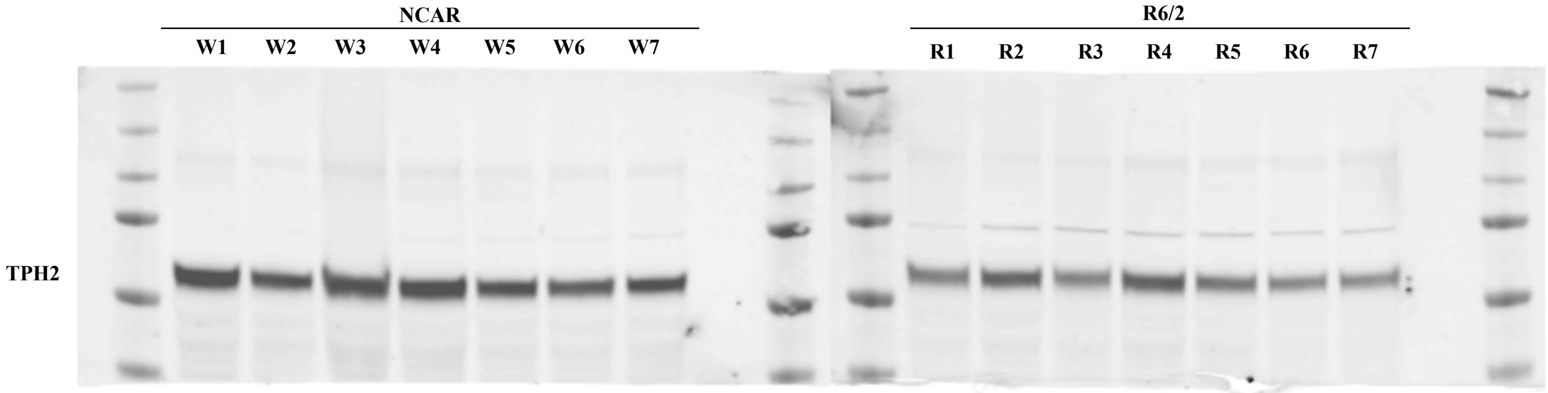
NCAR
W1 W2 W3 W4 W5 W6 W7
R6/2
R1 R2 R3 R4 R5 R6 R7
TPH2

I

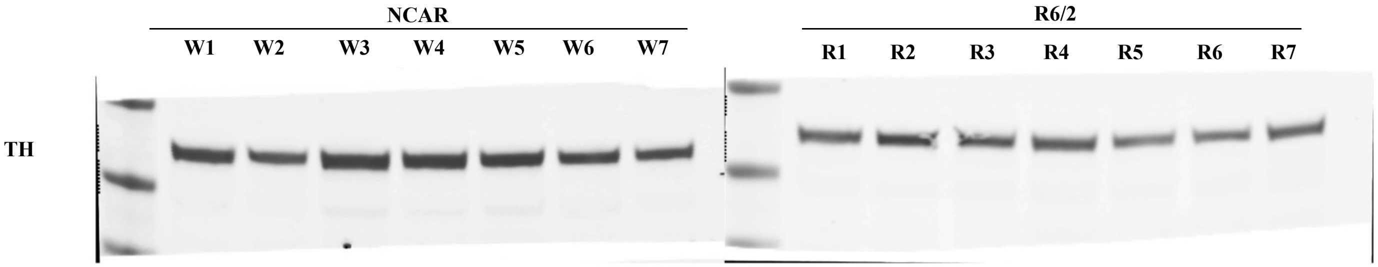
NCAR
W1 W2 W3 W4 W5 W6 W7
R6/2
R1 R2 R3 R4 R5 R6 R7
TH

J

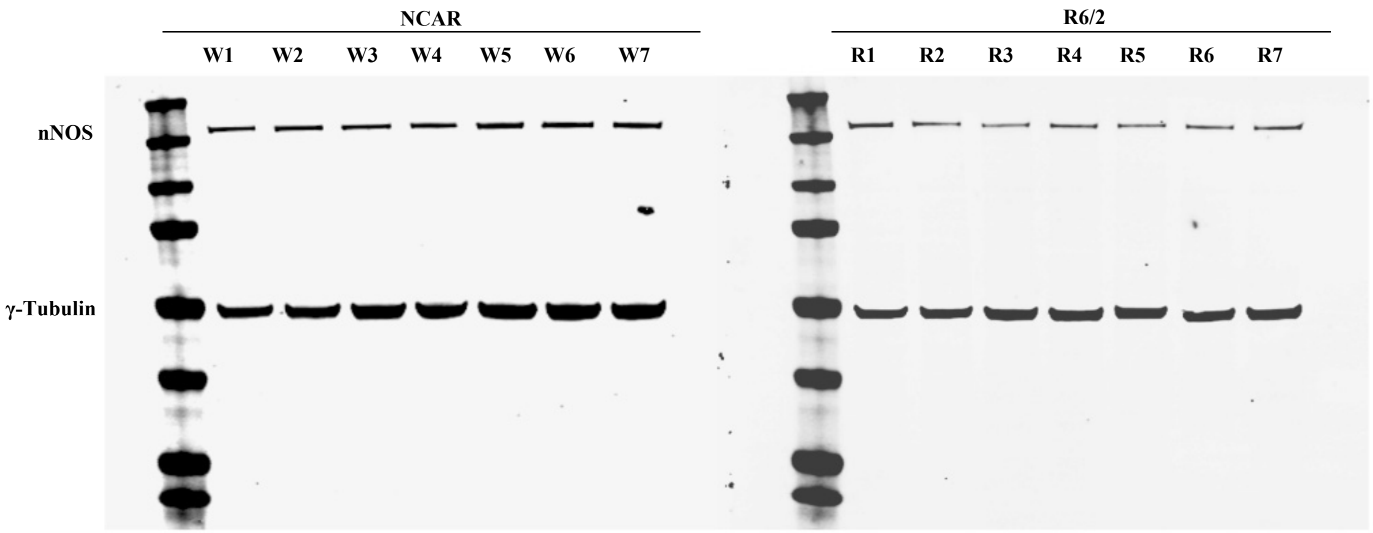

K

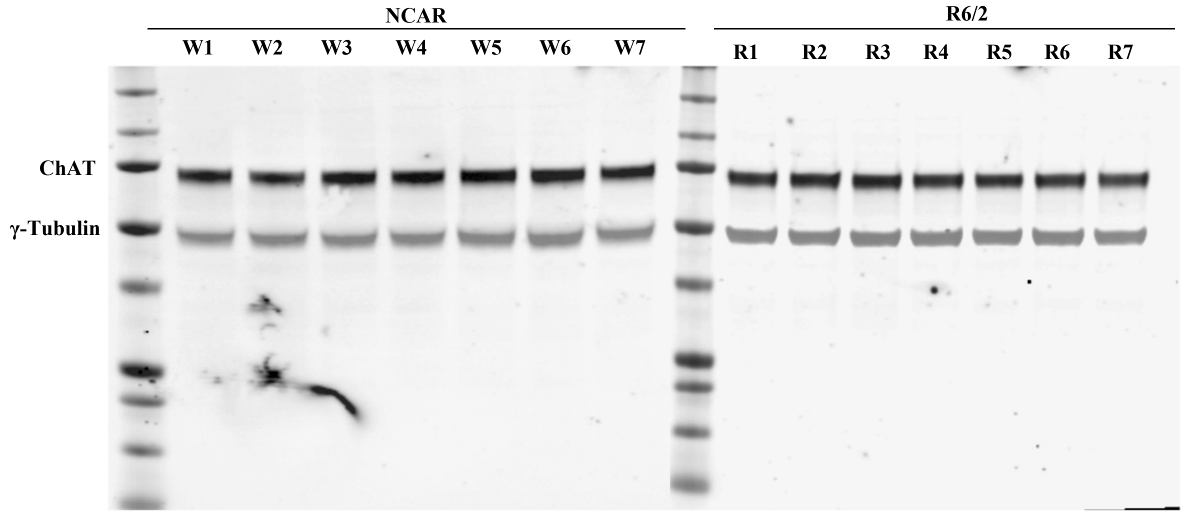

Fig. S11. Original blots of following 11 proteins before cropping (n = 7). (A) GTPCH. **(B)** DHFR. **(C)** QDPR. **(D)** MS. **(E)** MAT1/2A. **(F)** AHCY. **(G)** MTHFR. **(H)** TPH2. **(I)** TH. **(J)** nNOS. **(K)** ChAT.

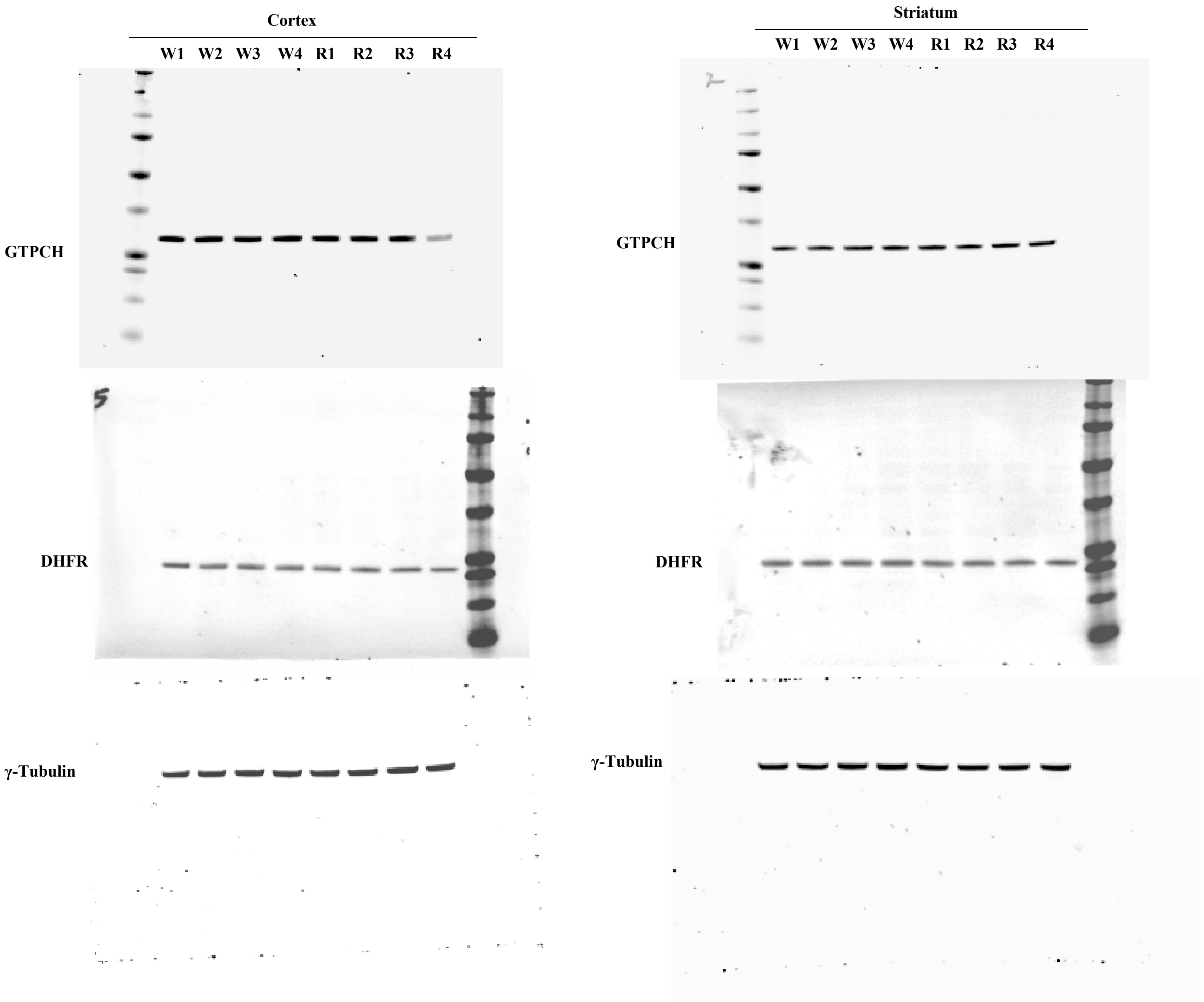

Fig. S12. Original blots of GTPCH, DHFR and γ-tubulin in cerebral cortex and striatum tissues of R6/2 and NCAR mice (n=4).
